# Detection of intercontinental reassortant H6 avian influenza viruses from wild birds in South Korea, 2015 and 2017

**DOI:** 10.3389/fvets.2023.1157984

**Published:** 2023-06-12

**Authors:** Ji-Yun Kim, Sun-Hak Lee, Da-Won Kim, Dong-Wook Lee, Chang-Seon Song, Dong-Hun Lee, Jung-Hoon Kwon

**Affiliations:** ^1^College of Veterinary Medicine, Kyungpook National University, Daegu, Republic of Korea; ^2^Avian Disease Laboratory, College of Veterinary Medicine, Konkuk University, Seoul, Republic of Korea; ^3^Wildlife Health Laboratory, College of Veterinary Medicine, Konkuk University, Seoul, Republic of Korea

**Keywords:** influenza virus, reassortment, phylogenetic analysis, wild bird, South Korea

## Abstract

Avian influenza viruses (AIVs) in wild birds are phylogenetically separated in Eurasian and North American lineages due to the separated distribution and migration of wild birds. However, AIVs are occasionally dispersed between two continents by migratory wild birds flying across the Bering Strait. In this study, we isolated three AIVs from wild bird feces collected in South Korea that contain gene segments derived from American lineage AIVs, including an H6N2 isolated in 2015 and two H6N1 in 2017. Phylogenetic analysis suggests that the H6N2 virus had American lineage matrix gene and the H6N1 viruses had American lineage nucleoprotein and non-structural genes. These results highlight that novel AIVs have continuously emerged by reassortment between viruses from the two continents. Therefore, continuous monitoring for the emergence and intercontinental spread of novel reassortant AIV is required to prepare for a possible future outbreak.

## 1. Introduction

Wild waterfowl are the natural hosts of avian influenza viruses (AIVs) (1) Because of the geographical barrier, AIVs are separated into two phylogenetic lineages, the Eurasian and American lineages (2). However, some migratory waterfowl (e.g., the Northern Pintail duck; *Anas acuta* and Greater White-fronted goose; *Anser albifrons*) move intercontinentally, causing genetic mixing between the two lineages of AIVs (3–8).

Previous surveillance studies on AIVs in wild birds provide evidence for the intercontinental exchange of AIVs. For example, the Eurasian H6 subtype AIV has been reported in North America since the 1990s and it replaced the prevailing North American H6 AIV (8). AIVs subtype H9N2, which contains six genes originating from North America, were simultaneously isolated in South Korea, China, and Alaska (5–7). In 2019, subtype H6N5 AIV carrying all eight gene segments from North American ancestors was detected in Mandarin duck in South Korea (4). In addition, the intercontinental spread of the highly pathogenic avian influenza (HPAI) virus from Eurasia to North America was detected in 2014 and 2021 (9, 10). Western Alaska, where migratory flyways of waterfowl overlap, is the location to encounter AIVs from Eurasian and North American lineages (3).

In the current study, we report three reassortant H6 viruses containing gene segments originating from North America. Complete genome sequencing and phylogenetic analysis were used to find the origin of each gene segment.

## 2. Materials and methods

We collected fresh fecal samples from wild bird habitats in South Korea for routine AIV surveillance during the fall migration and wintering seasons of wild waterfowl. In the 2015–2016 winter, we collected total of 1,896 fecal samples and in the 2016–2017 winter, we collected total of 8,096 fecal samples. Following as the previous study, fecal samples were examined for influenza A virus by real-time reverse transcription polymerase chain reaction (rRT-PCR) targeting the matrix (*M*) gene (11). Virus isolation was done using embryonated SPF chicken eggs. We found three reassortant H6 viruses containing gene segments originating from the American continent: A/Mandarin duck/Korea/K15-68/2015 (H6N2), A/Greater White-fronted goose/Korea/K16-727-5/2017 (H6N1), and A/Greater White-fronted goose/Korea/K16-738/2017 (H6N1) (designated as K15-68, K16-727-5, and K16-738, respectively). The date and location information of the isolated viruses is shown in Table 1. We sequenced full-length genomes of the isolates using the Illumina MiSeq system. We deposited the nucleotide sequences of each virus into the Global Initiative for Sharing All Influenza Data (GISAID) database (accession nos. EPI_ISL_11110143, EPI_ISL_11112483, and EPI_ISL_11112543, respectively). The host of each fecal sample positive for AIV was identified using a DNA barcoding technique, as previously described (12).

**Table 1:** Summary of viruses used in this study.

Virus name	GISAID accession #	Collection date	Province	Location coordinate
A/Mandarin duck/Korea/K15-68/2015(H6N2)	EPI_ISL_11110143	2015-11-11	Cheonsu bay	36°36’21’’N 126°25’9’’E
A/Greater white-fronted goose/Korea/K16-727-5/2017(H6N1)	EPI_ISL_11112483	2017-03-14	Ganghwa-gun	37°44′50″N 126°29′8″E
A/Greater white-fronted goose/Korea/K16-738/2017(H6N1)	EPI_ISL_11112543	2017-03-14	Ganghwa-gun	37°44′50″N 126°29′8″E

Comparative phylogenetic analysis of each gene was conducted to trace their origin. For phylogenetic analysis, all available sequences collected between 2010 and 2021 were downloaded from GISAID. To prevent the omission of intercontinental spread viruses during the subsampling process, we classified all downloaded sequences into two groups before subsampling: one containing the viruses isolated from Asia, Africa, and Europe and the other containing the viruses isolated from South and North America. We reduced the number of sequences in each gene segment of each group based on sequence identities of 97 ~ 99% using the program CD-HIT (13). Maximum-likelihood (ML) phylogenetic trees were constructed using a general time reversible (GTR) substitution model with 500 bootstrap replications in RAxML version 8.2.11 (14). We used BLAST^1^ to search for sequences with the highest identity to each virus for each gene segment.

To verify the result of the phylogenetic tree, we constructed time-scaled phylogenetic trees using BEAST version 1.10.4 (15). The GTR nucleotide substitution model and uncorrelated lognormal relaxed molecular clock model were used for constructing a time-scaled maximum clade credibility (MCC) tree. MCC trees were visualized using FigTree 1.4.2.^2^

To evaluate the pathogenicity of the viruses in chickens, a total of 15 three-week-old SPF chickens (Namdeok SPF, Korea) were divided into 3 groups (5 chickens/group). The chickens were inoculated with H6 LPAI viruses at 10^6.0^ EID_50_ in a volume of 100ul by intranasal route. At 2-, 3-, 5-, and 7-days post-infection (dpi), oropharyngeal (OP) and cloacal (CL) swabs were collected from all chickens and examined for virus shedding using a quantitative real-time reverse transcription polymerase chain reaction (rRT-PCR) targeting the matrix gene, as described previously (11). For each virus, the standard curve was used to convert the Ct values into equivalent EID_50_ titer. All chickens were monitored daily for clinical signs and mortality. Serum samples were collected for serological investigations including anti-NP ELISA (Bionote, Inc., Korea) and Hemagglutination inhibition (HI) testing for homologous HA-specific antibodies.

## 3. Results

We successfully obtained complete genome sequences of the K15-68, K16-727-5, and K16-738 viruses. Host species of K15-68 was identified as Mandarin duck, and K16-727-5 and K16-738 viruses’ host were identified as Greater White-fronted goose using DNA barcoding. The genome sequences of K16-727-5 and K16-738 which were isolated on the same sample collection day were almost identical (NP: 99.936%, NS: 99.944%, and other 6 genes: 100%).

**Figure 1:**
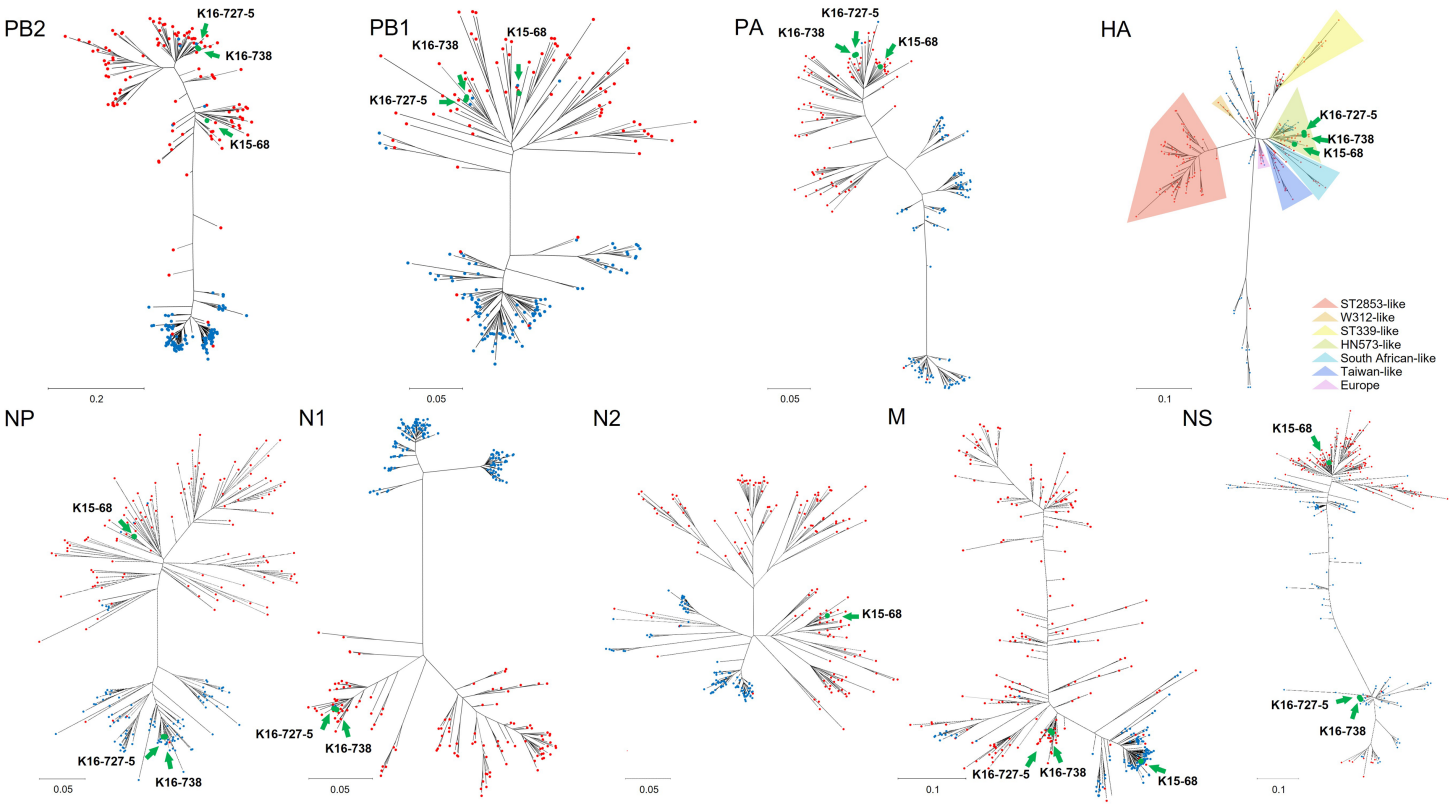
Maximum-likelihood phylogenetic trees of each gene segment of avian influenza virus including Eurasian lineage and American lineage genome sequences. Red and blue circles indicate lineages of Eurasia and America, respectively. Green circles indicate genome sequences of novel viruses which isolated in this study; K15-68, K16-727-5, K16-738. Maximum-likelihood phylogenetic trees for each gene segment are shown in Supplementary Figure S1.

BLAST research indicated that the matrix (M) gene of K15-68 strain shared >99% nucleotide identity with the Guatemalan origin H14N3 subtype AIV. The nucleoprotein (NP) gene of K16-727-5 and K16-738 strains sharing 99.94% nucleotide identity with H9N6 subtype AIV isolated from Missouri, United States, and the nonstructural (NS) gene shared 98.31% nucleotide identity with Ohio, USA isolate (Table 2). All other segments showed >98% identity with the low-pathogenicity AIVs (LPAI) identified in South Korea, Japan, China, and Mongolia. Corresponding to the BLAST results, the ML and Bayesian phylogenetic analysis showed that the M gene of K15-68 and the NP and NS gene of K16-727-5 and K16-738 clustered with the American lineage wild bird AIVs and the rest of the gene segments were clustered with the Eurasian lineage wild bird AIVs (Figure 1; Supplementary Figures S1, S2). These results demonstrate that the three viruses were generated by reassortment between Eurasian and American AIVs.

**Table 2:** BLAST results of 8 gene segments of three viruses isolated in this study.

Virus	Gene	BLAST results		
		Strain	% Genetic identity	GenBank accession #
K15-68	PB2	A/duck/Hokkaido/201/2014(H1N1)	99.01%	LC339528.1
	PB1	A/waterfowl/Korea/S353/2016(H11N9)	99.83%	KX703017.1
	PA	A/waterfowl/Korea/S245/2016(H6N2)	99.60%	KX761368.1
	HA	A/wild bird/Jiangxi/P419/2016(H6N8)	98.34%	KX867857.1
	NP	A/waterfowl/Korea/S245/2016(H6N2)	99.74%	KX761370.1
	N2	A/duck/Miyazaki/CAD-1/2016(H4N2)	99.38%	LC415036.1
	**M**	**A/blue-winged teal/Guatemala/CIP049H105–15/2011(H14N3)**	**99.42%**	**KJ195679.1**
	NS	A/waterfowl/Korea/S245/2016(H6N2)	99.44%	KX761373.1
K16-727-5	PB2	A/waterfowl/Korea/S245/2016(H6N2)	99.36%	KX761366.1
	PB1	A/duck/Akita/51019/2017(H5N3)	98.88%	MK592459.1
	PA	A/Duck/Mongolia/782/2017(H7N3)	99.01%	MH744642.1
	HA	A/duck/Hunan/10.27_YYGK57B2-O/2016(mixed)	99.42%	MW108112.1
	**NP**	**A/northern shoveler/Missouri/17OS4858/2017(H6N2)**	**99.94%**	**MK237594.1**
	N1	A/wild waterfowl/Korea/F14-5/2016(H6N1)	99.65%	MH130116.1
	M	A/mallard/Netherlands/89/2017(H4N6)	99.42%	MK192396.1
	**NS**	**A/Mallard/Ohio/18OS1894/2018(mixed)**	**98.31%**	**MT565511.1**
K16-738	PB2	A/waterfowl/Korea/S245/2016(H6N2)	99.36%	KX761366.1
	PB1	A/duck/Akita/51019/2017(H5N3)	98.88%	MK592459.1
	PA	A/Duck/Mongolia/782/2017(H7N3)	99.01%	MH744642.1
	HA	A/duck/Hunan/10.27_YYGK57B2-O/2016(mixed)	99.42%	MW108112.1
	**NP**	**A/northern shoveler/Missouri/17OS4858/2017(H6N2)**	**99.94%**	**MK237594.1**
	N1	A/wild waterfowl/Korea/F14-5/2016(H6N1)	99.65%	MH130116.1
	M	A/mallard/Netherlands/89/2017(H4N6)	99.42%	MK192396.1
	**NS**	**A/Mallard/Ohio/18OS1894/2018(mixed)**	**98.31%**	**MT565511.1**

The American lineage strains were highlighted in bold.

K15-68, A/Mandarin duck/Korea/K15-68/2015(H6N2); K16-727-5, A/Greater white-fronted goose/Korea/K16-727-5/2017 (H6N1); K16-738, A/Greater white-fronted goose/Korea/K16-738/2017(H6N1). PB, polymerase basic protein polymerase basic gene; PA, polymerase acidic gene; HA, hemagglutinin gene; NP, nucleoprotein gene; NA, neuraminidase gene; M, matrix protein; NS, nonstructural protein.

The HA genes from H6N1 and H6N2 AIVs have some phylogenetic distance. Eurasian lineage H6 genes are divided into seven groups; ST339-like, W312-like, ST2853-like, HN573-like, South African-like, Taiwan-like, and Europe (Figure 1) (16). HN573-like is divided into three subgroups, subgroup 1 (mixed group), 2 (Eurasian group), and 3 (American group). All three isolates cluster with HN573-like, but with different subgroups (Supplementary Figure S1D). H6N2 belongs to subgroup 1 and H6N1 belongs to subgroup 2.

The amino acid changes related to the mammalian adaptation, including Q591K, E627K, and D701N mutations in PB2 (17), N137/ E190V/ G228S triad, and Q226L mutation in HA (H3 numbering) (18, 19), were not detected in the viruses isolated in this study. The NA stalk deletion, which has been associated with adaptation to gallinaceous hosts (20), was also not detected.

Three-week-old SPF chickens were inoculated with the H6 viruses to study their infectivity and virulence. During the 14-day experiment, no obvious clinical signs and no mortality were observed. Virus shedding through the OP and CL routes was detected during 3–7 dpi, but the detected amount of viruses was very low (<10^3.5^ EID_50_) (Table 2). For the chickens inoculated with K15-68 and K16-727-5 virus, mean shedding titers for both routes did not exceed 10^1.0^EID_50_ throughout the whole period of the experiment. Chickens inoculated with K16-738 showed a peak of OP shedding at 3 dpi and a peak of CL shedding at 7 dpi, with mean shedding titers of 10^1.85^ EID_50_ and 10^1.84^ EID_50_, respectively. Both HI and NP-specific antibodies were detected in two of five, one of five, and one of five chickens inoculated with K15-68, K16-727-5, and K16-768, respectively (Table 3).

**Table 3:** Viral shedding and antibody responses of chickens inoculated with H6 avian influenza viruses.

viruses	No. of positive chickens/total chickens (Mean viral titer, log_10_ EID_50_/ml ± SD)^c^								Serology, no. of positive/total^f^	
	OP^a^				CL^b^				NP-ELISA^d^	HI Assay^e^
	2 dpi	3 dpi	5dpi	7 dpi	2 dpi	3 dpi	5dpi	7 dpi	(Mean PI)	(Mean Titer)
K15-68 (H6N2)	0/5	3/5 (0.8 ± 0.2)	4/5 (0.8 ± 0.2)	5/5 (0.6 ± 0.4)	0/5	0/5	3/5 (0.6 ± 0.2)	2/5 (0.7 ± 0.0)	2/5 (40.0)	2/5 (2^4^)
K16-727-5 (H6N1)	0/5	1/5 (1.5)	2/5 (0.9 ± 0.2)	5/5 (0.5 ± 0.0)	0/5	0/5	2/5 (0.7 ± 0.1)	4/5 (1.0 ± 0.3)	1/5 (11.6)	1/5 (2^3^)
K16-738 (H6N1)	0/5	4/5 (2.0 ± 0.3)	3/5 (1.8 ± 0.5)	4/5 (1.5 ± 0.0)	1/5 (1.8)	2/5 (1.3 ± 0.1)	3/5 (2.1 ± 0.8)	3/5 (2.2 ± 1.0)	1/5 (23.4)	1/5 (2^7^)

a OP: Oropharyngeal swab.

b CL: Cloacal swab.

c Postive birds were indicated by real-time RT-PCR. Ct-value < 36 was considered as positive. Mean viral titer and standard deviation were calculated after converting the Ct values into equivalent EID_50_ titer by using the standard curves for each virus.

d Anti-influenza A nucleoprotein (NP)-specific antibody was analyzed using the commercially available multispecies competitive NP-ELISA Kit (Bionote, Korea). A percent inhibition (PI) value >50 was regarded as positive.

e HI assay: hemagglutination inhibition assay. An HI titer ≥4 was regarded as positive.

f Serum samples were collected from the birds at 14 days after the infection.

## 4. Discussion

In the current study, we report American-Eurasian reassortants H6N1 and H6N2 viruses isolated from two wild bird species, the Greater White-fronted goose and Mandarin duck. Since the first report of North American-Eurasian reassortant AIVs detected from wild birds in South Korea in 2010 (21), North American lineage AIV gene segments have been detected continuously in South Korea (4, 21–23). Previous studies also detected multiple Eurasian lineage AIV genes in Alaska, indicating the bi-directional flow of AIV exchange between East Asia and North America (24).

Recently, intercontinental reassortants AIVs have been frequently detected (4, 6, 9, 21–23, 25, 26). The reason for the frequent discovery of intercontinental reassortants is not fully determined yet. We assume that climate change can be a factor in contributing to frequent exchange of AIVs between two continents. A previous study indicated that an abnormal climate in Africa might have contributed to the trans-continental introduction of HPAI H5Nx in Africa by migratory birds (27). The temperature of Alaska has increased since 2014 and it might affect the migration pattern of migratory birds and the ecology of AIVs in wild birds (28). On the other hand, Next-Generation Sequencing techniques have been widely used for AIV sequencing (23), and this high-throughput sequencing system would also contribute to the frequent detections of novel reassortants.

The reassortment of AIVs causes rapid changes in the biological characteristics of viruses and it could be a considerable threat to the poultry industry and public health (29). In 2014, in China, for example, human infection of AIV subtype H7N9 occurred by reassortment of AIVs in poultry and wild birds (30). In 2013, a case of human infection with subtype H6N1, AIV generated by reassortment carrying N137/E190V/G228S triad amino acid changes in HA, was reported in Taiwan (31). Although no mammalian adaptation mutation was detected in this study, the emergence of novel AIVs reassortants and the detection of mutation related to mammalian adaptation highlights the importance of wild bird surveillance in terms of the ‘One Health’ concept.

The viruses isolated in this study showed limited infectivity in chickens similar to other wild bird-origin H6 viruses tested in previous studies (32–35). These H6 viruses may be poorly adapted to chickens, but we assume that they possibly replicate in domestic waterfowl without prior adaptation which could lead to the spread and maintenance of H6 viruses in land-based poultry. Bahl et al. showed that the introduction and establishment of Eurasian H6 viruses in North America dramatically changed the evolutionary dynamics of the influenza virus in wild birds (36). Thus, genomic surveillance and *in vivo* pathobiology studies should be continued to monitor the evolution of reassortant H6 viruses and their host ranges.

Constant monitoring for AIVs in wild birds is essential to detect the introduction of new viruses and trace the dispersion path. Particularly, as a major wintering site for various wild birds (37), South Korea would be an important location for monitoring the intercontinental exchange of AIVs. Combined with recently developed sequencing technology, continuing monitoring of the intercontinental dispersion of AIVs would expand our knowledge of AIV ecology and epidemiology.

## Data availability statement

The datasets presented in this study can be found in online repositories. The names of the repository/repositories and accession number(s) can be found in the article/Supplementary material.

## Ethics statement

The animal study was reviewed and approved by Institutional Animal Care and Use Committee of Konkuk University.

## Author contributions

C-SS, D-HL, and J-HK contributed to conception and design of the study. S-HL collected and analyzed samples and conducted an animal experiment. J-YK, D-WK, and D-WL organized data and performed genetic analysis. J-YK visualized data and wrote the first draft of the manuscript. S-HL wrote animal experiment sections. D-HL and J-HK revised final manuscript. All authors contributed to the article and approved the submitted version.

## Funding

This research was funded by Kyungpook National University Research Fund, 2020.

## Conflict of interest

The authors declare that the research was conducted in the absence of any commercial or financial relationships that could be construed as a potential conflict of interest.

## Publisher’s note

All claims expressed in this article are solely those of the authors and do not necessarily represent those of their affiliated organizations, or those of the publisher, the editors and the reviewers. Any product that may be evaluated in this article, or claim that may be made by its manufacturer, is not guaranteed or endorsed by the publisher.

## Footnotes

^1^https:///blast.ncbi.nlm.nih.gov/Blast.cgi

^2^http://tree.bio.ed.ac.uk/software/figtree/
