# Supplementary material for: Detection of intercontinental reassortant H6 avian influenza viruses from wild birds in South Korea, 2015 and 2017
Source: Front Vet Sci. 2023 Jun 12;10:1157984. doi: 10.3389/fvets.2023.1157984 (PMC10291271; doi:10.3389/fvets.2023.1157984)
Supplement: Supplementary file 1 [file Data_Sheet_1.docx]

Supplementary Material


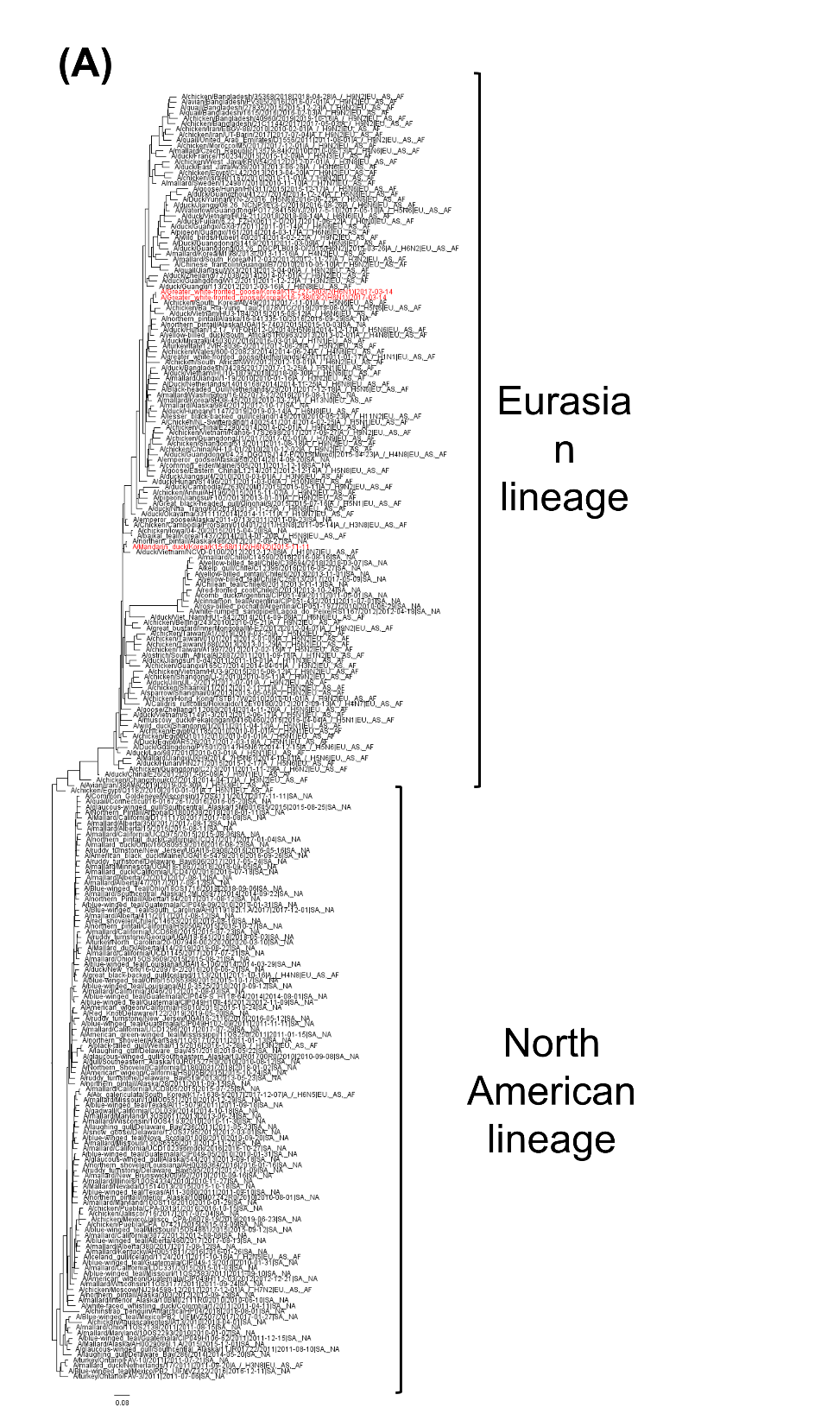


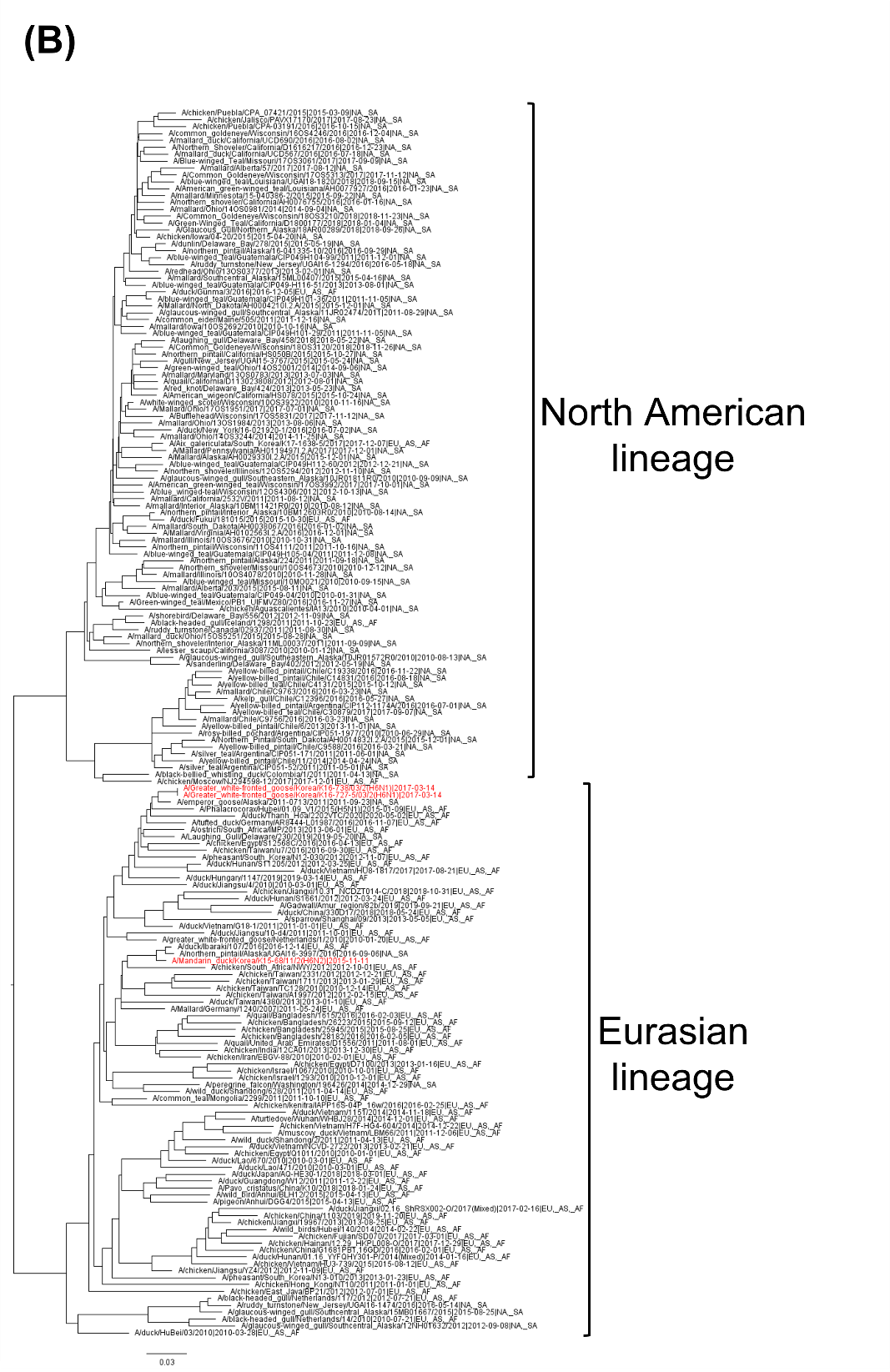


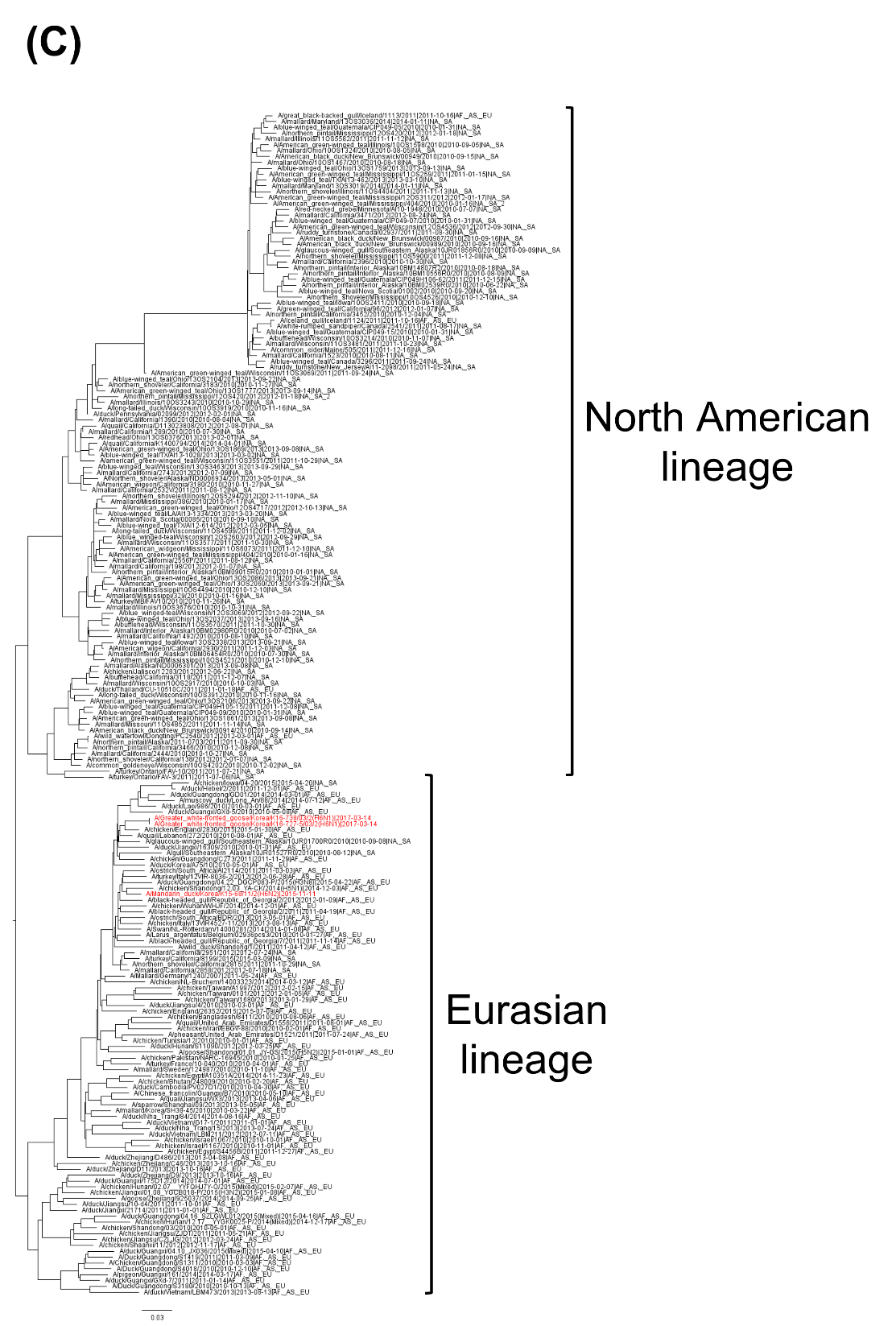


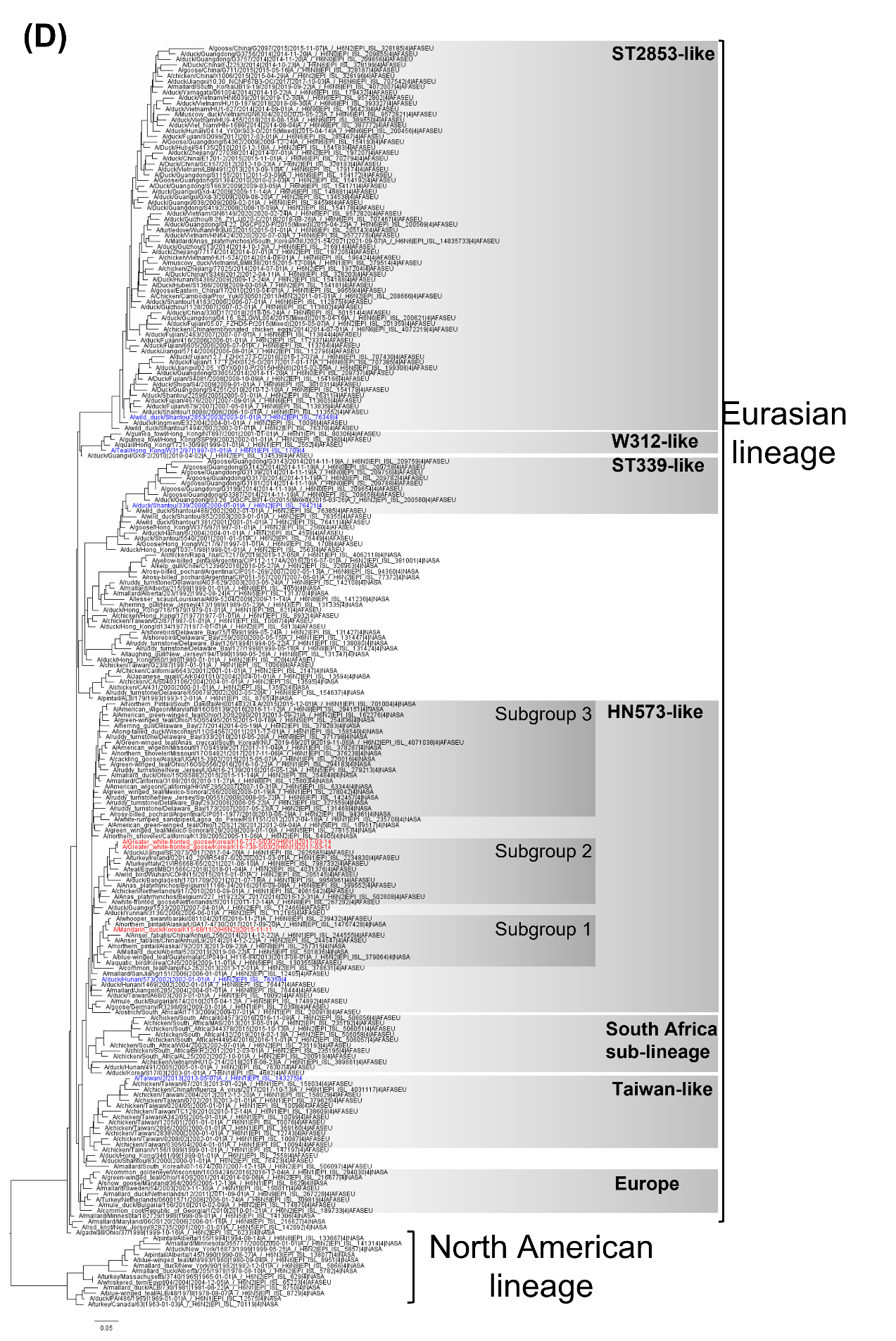


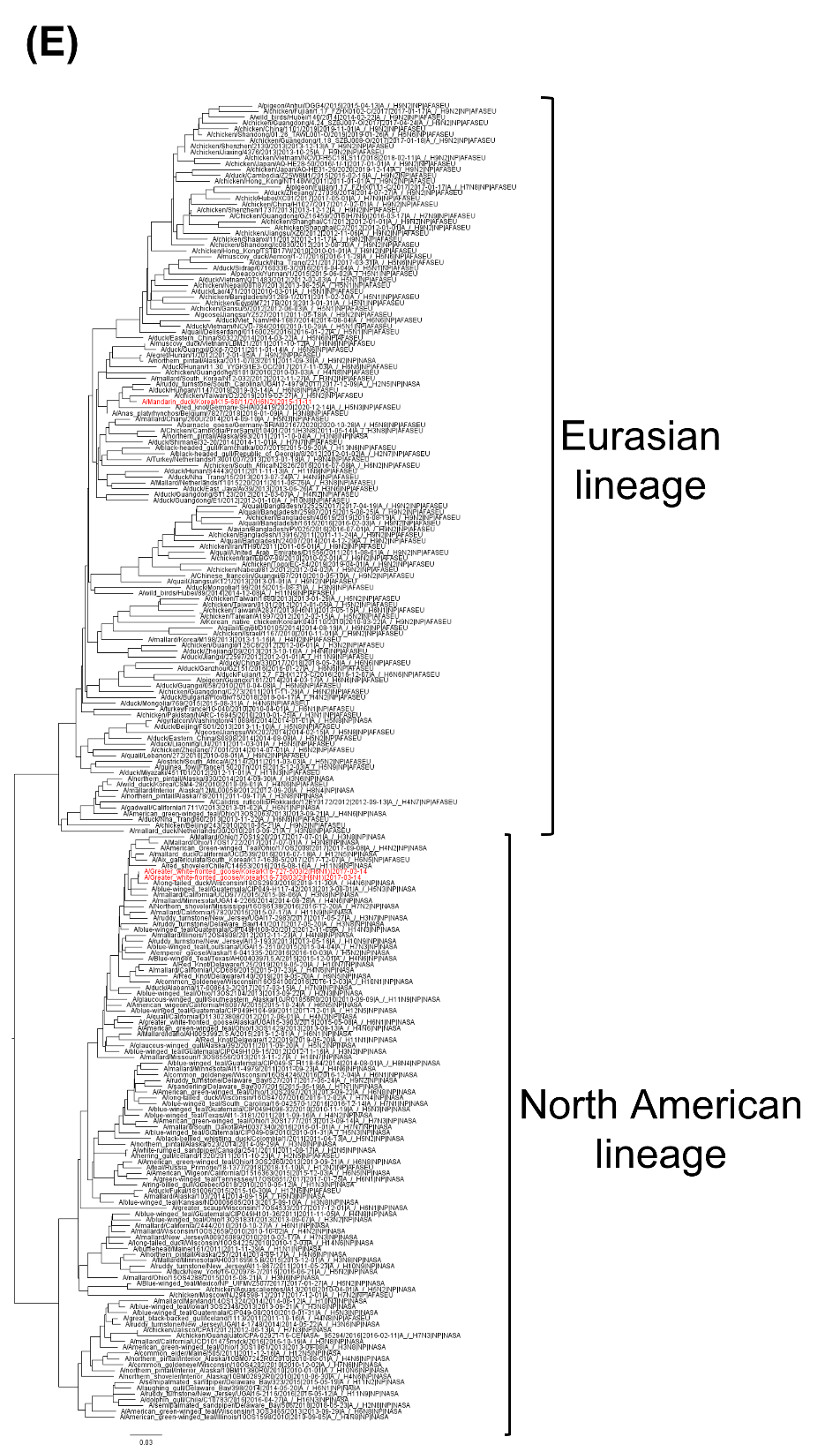


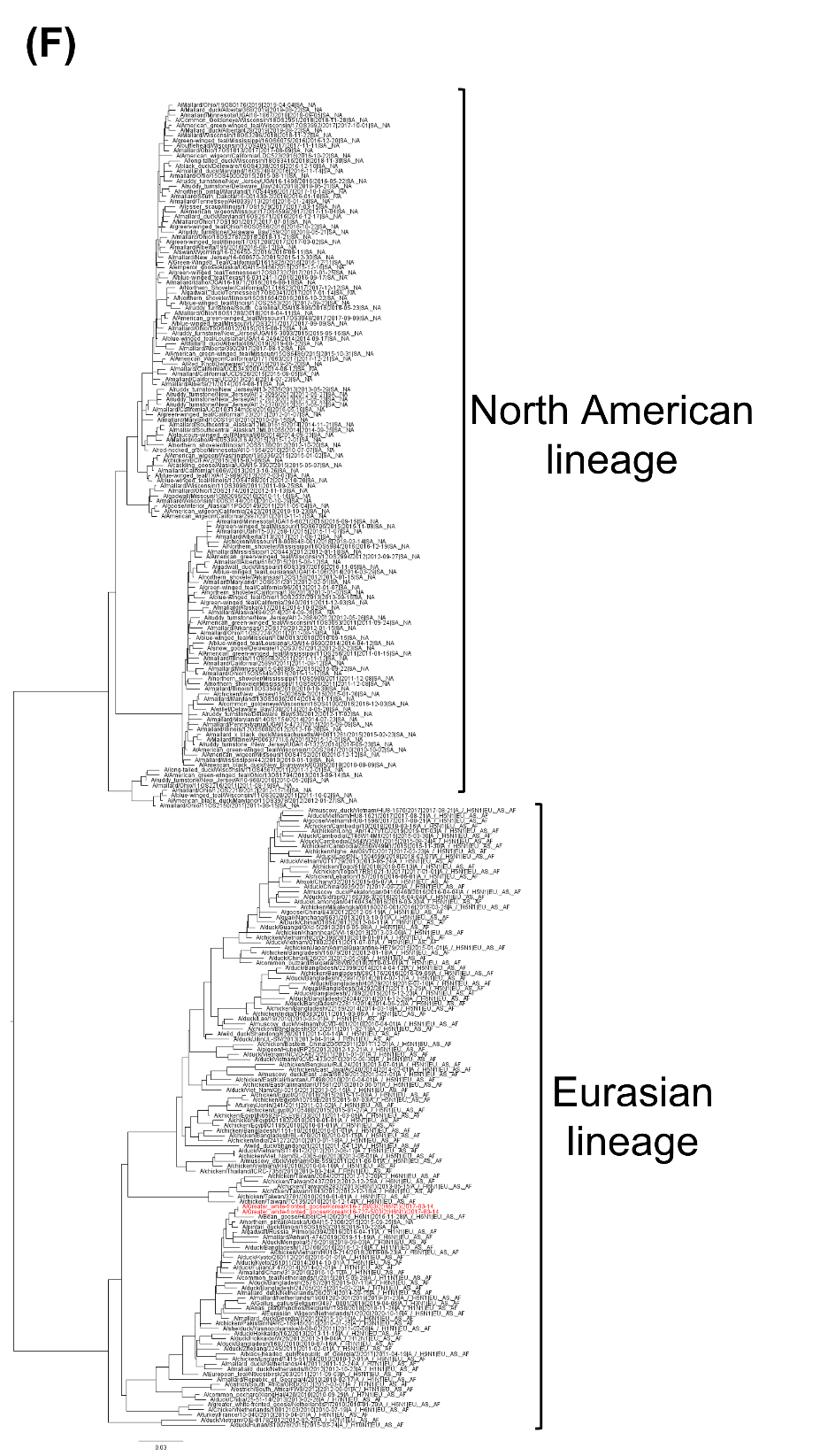


**
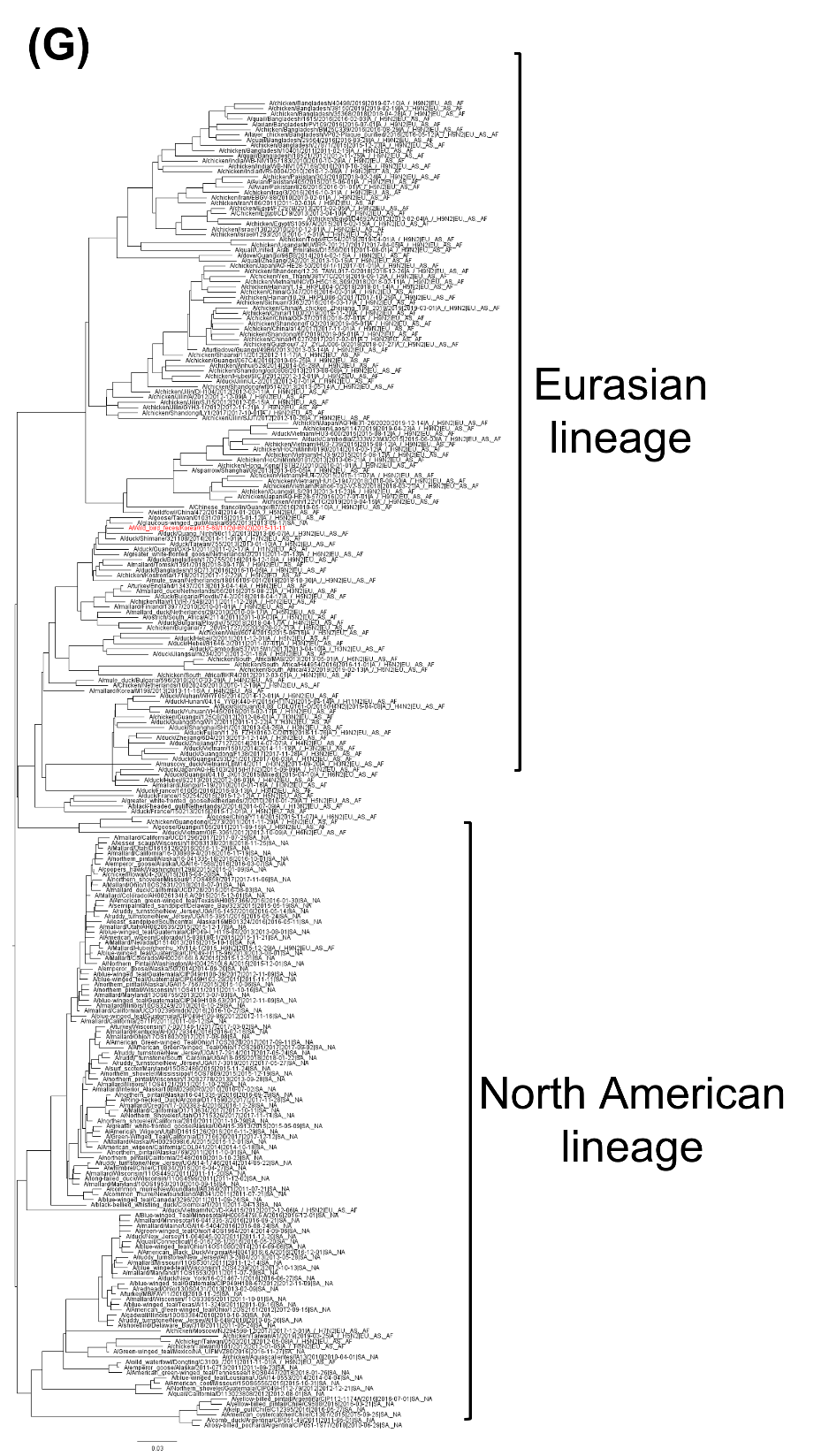
**

**
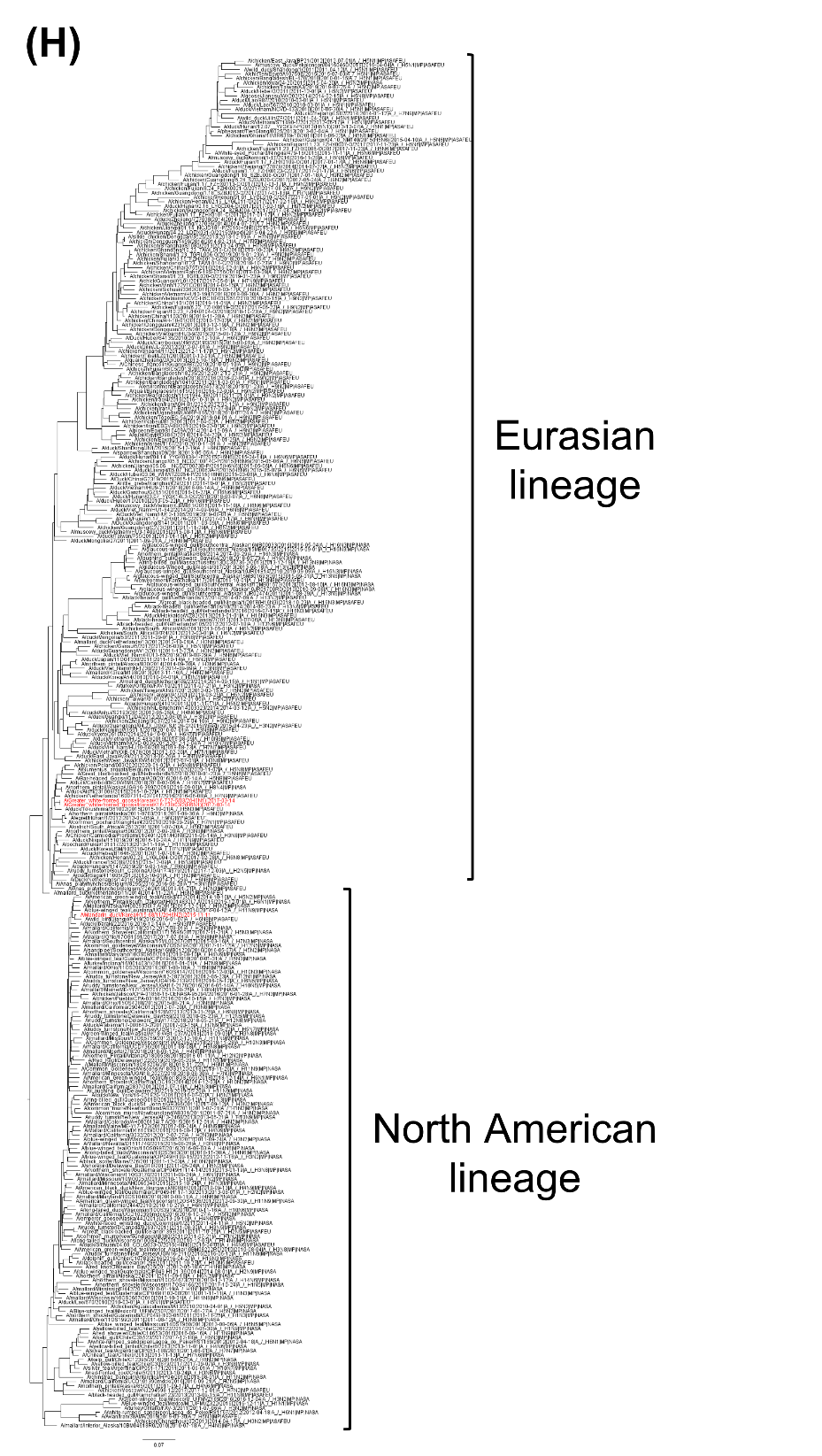
**

**
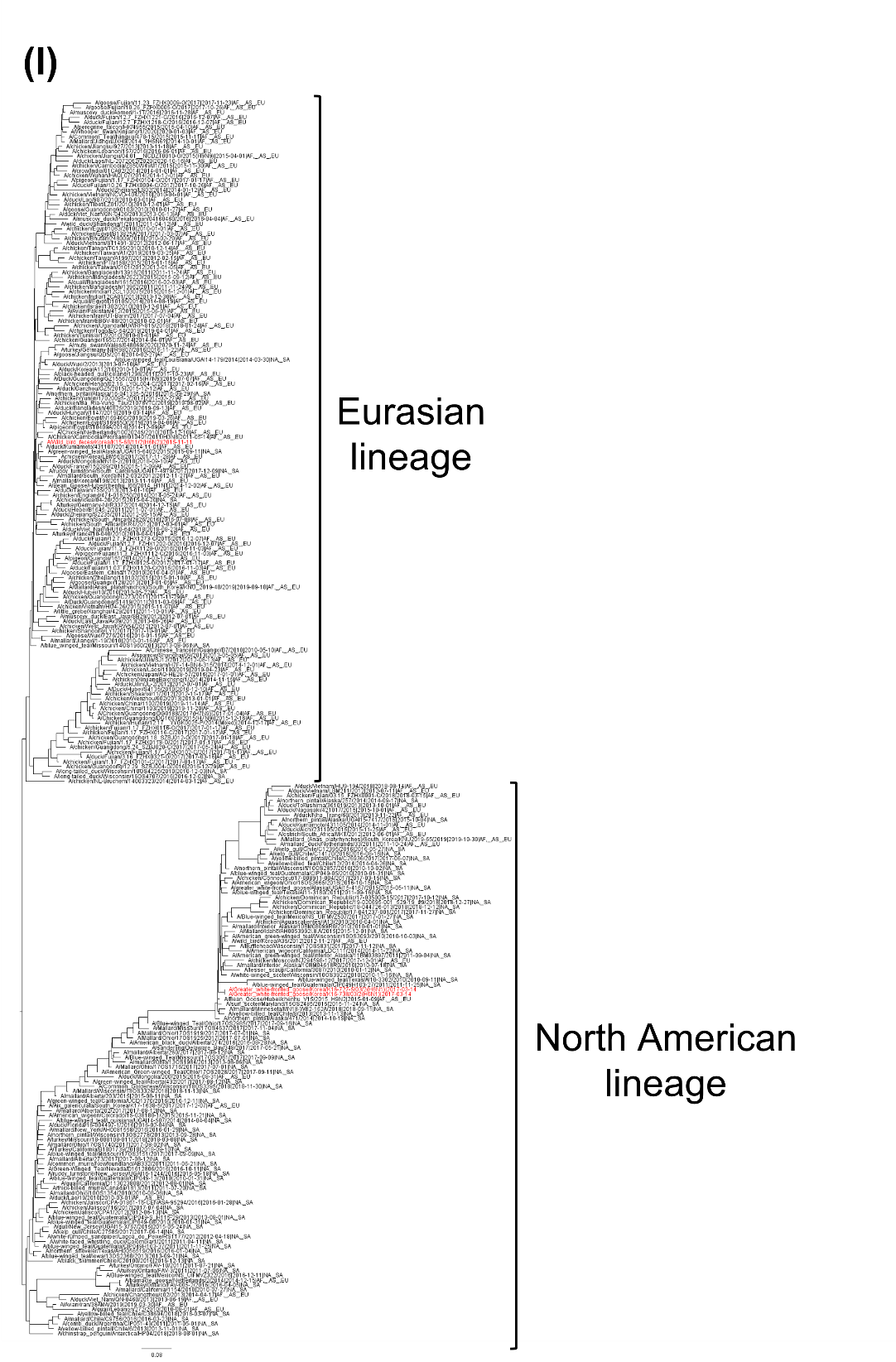
**

**Supplementary Figure 1.** Maximum-likelihood phylogenetic trees of each gene segment.

Three novel viruses isolated in this study indicated by red text. (A) Polymerase basic 2 gene; (B) polymerase basic 1 gene; (C) polymerase acidic gene; (D) hemagglutinin gene; (E) nucleoprotein gene; (F) neuraminidase subtype N1 gene; (G) neuraminidase subtype N2 gene; (H) matrix gene; (I) nonstructural gene.


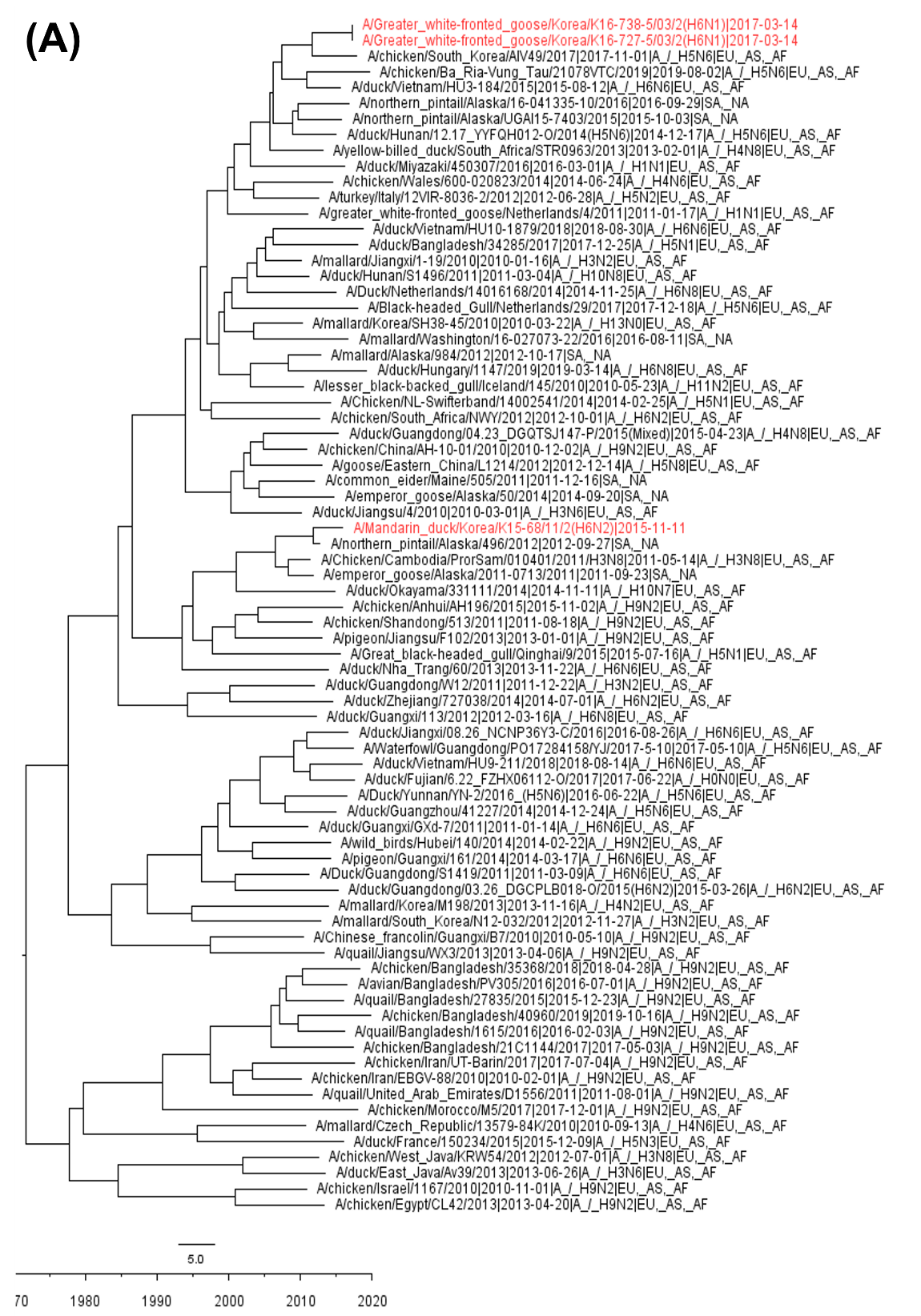


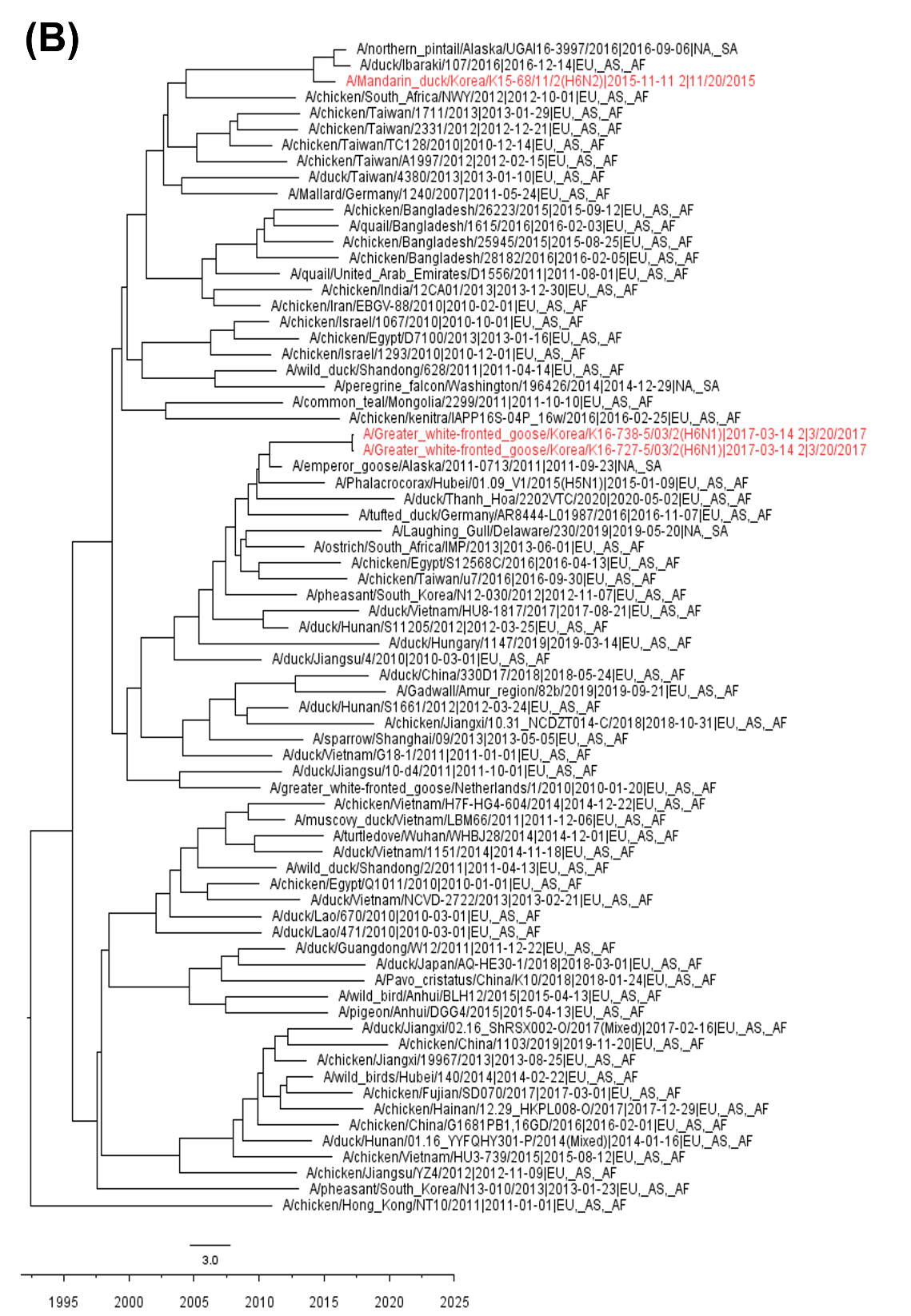


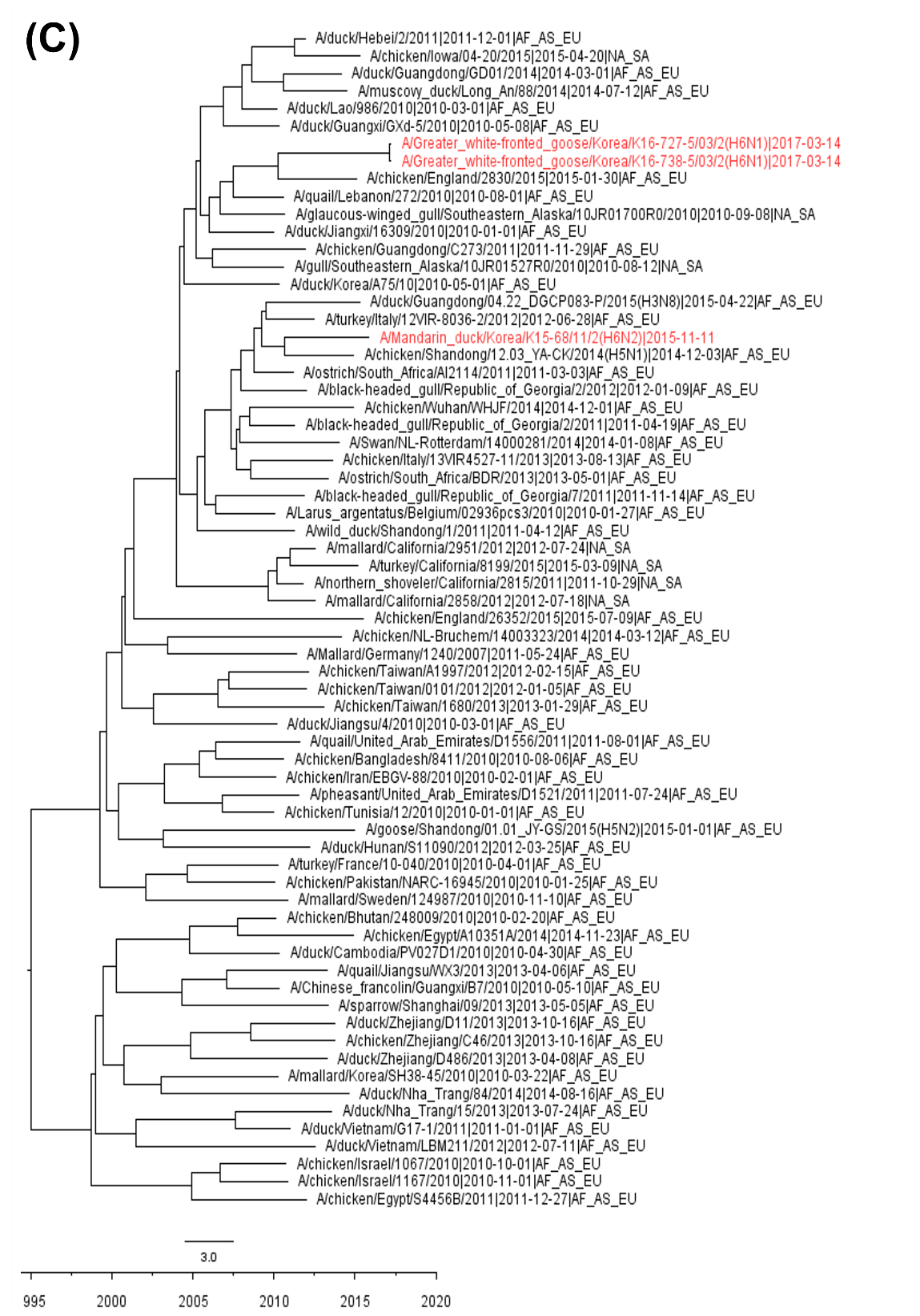


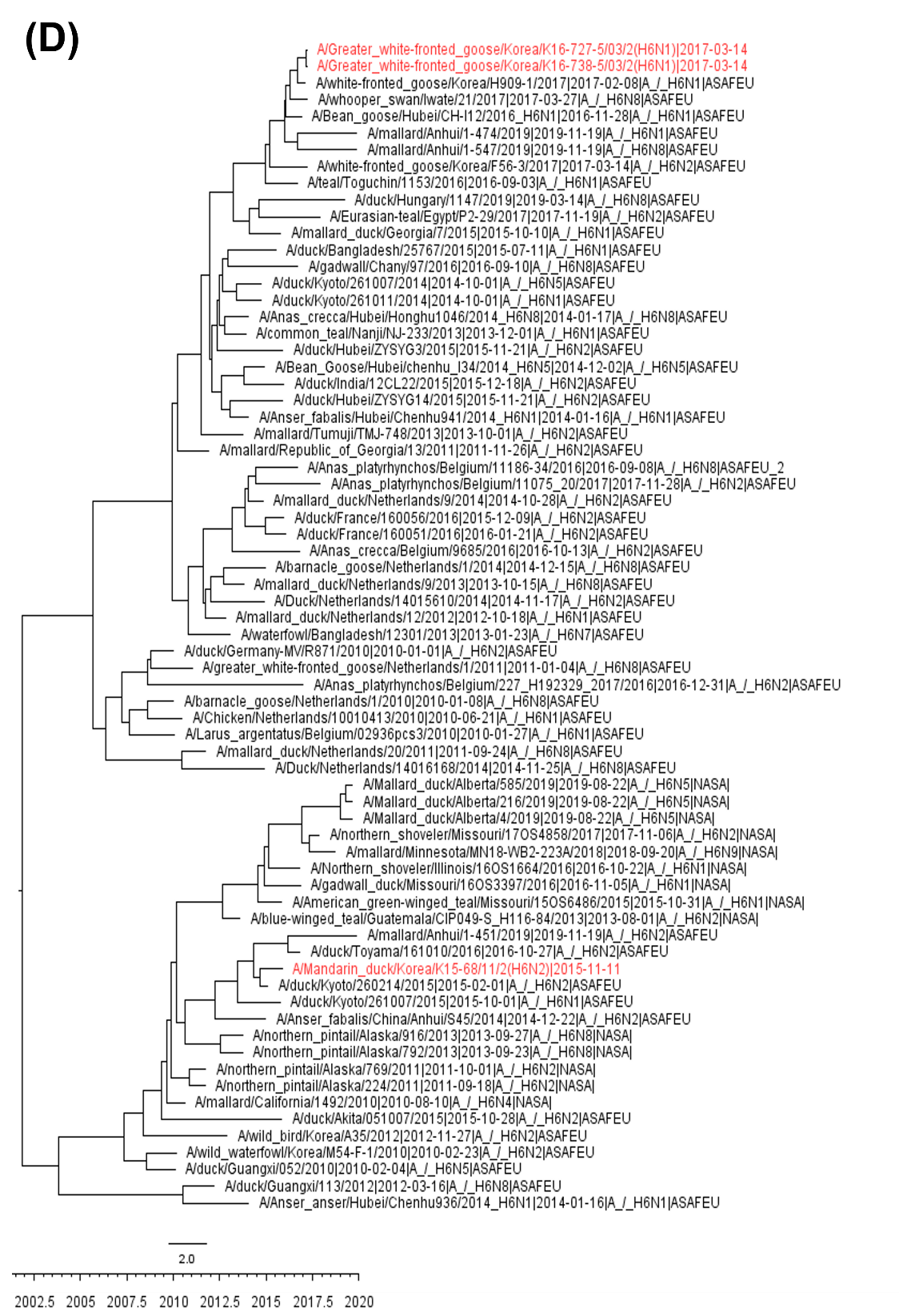


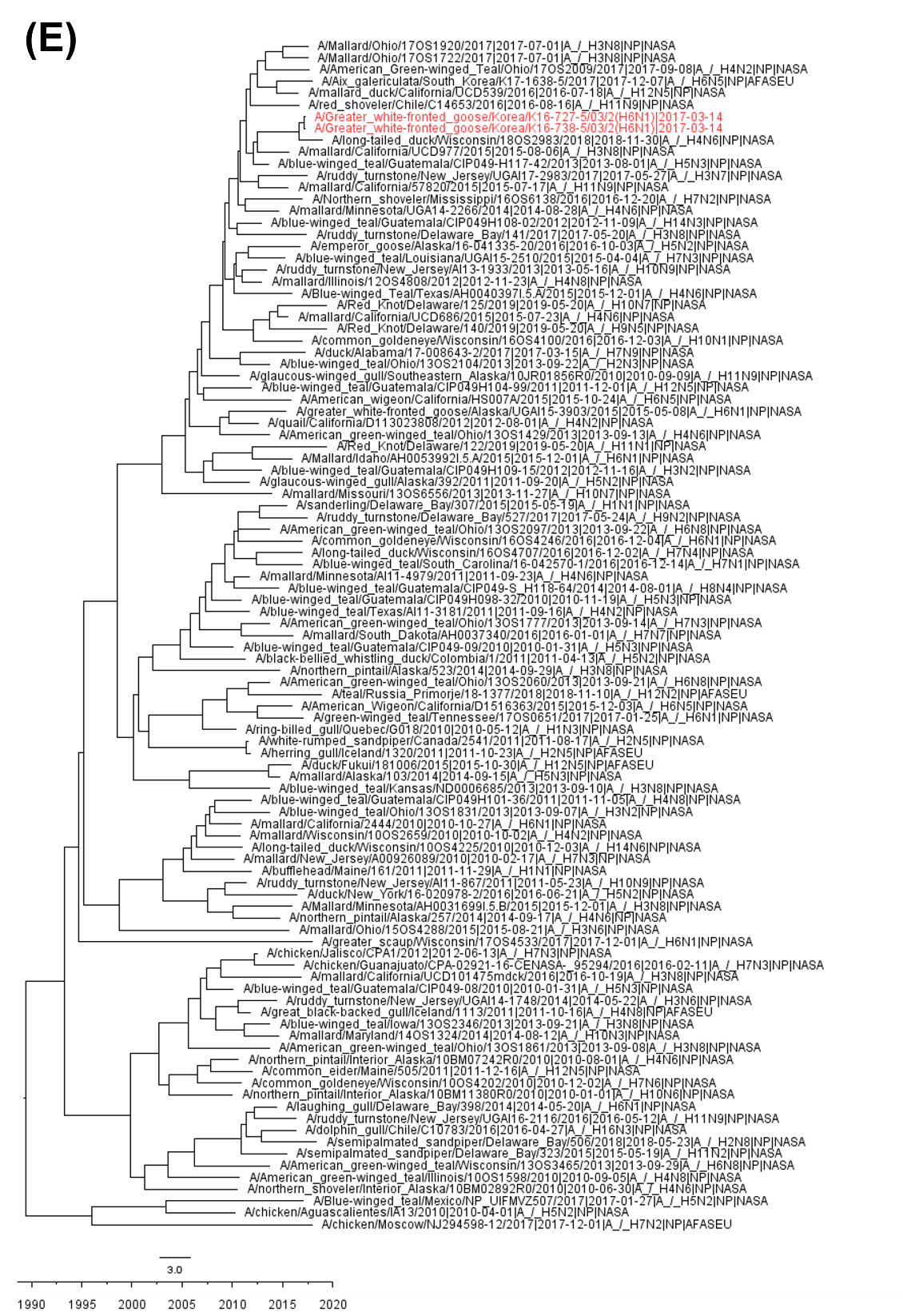


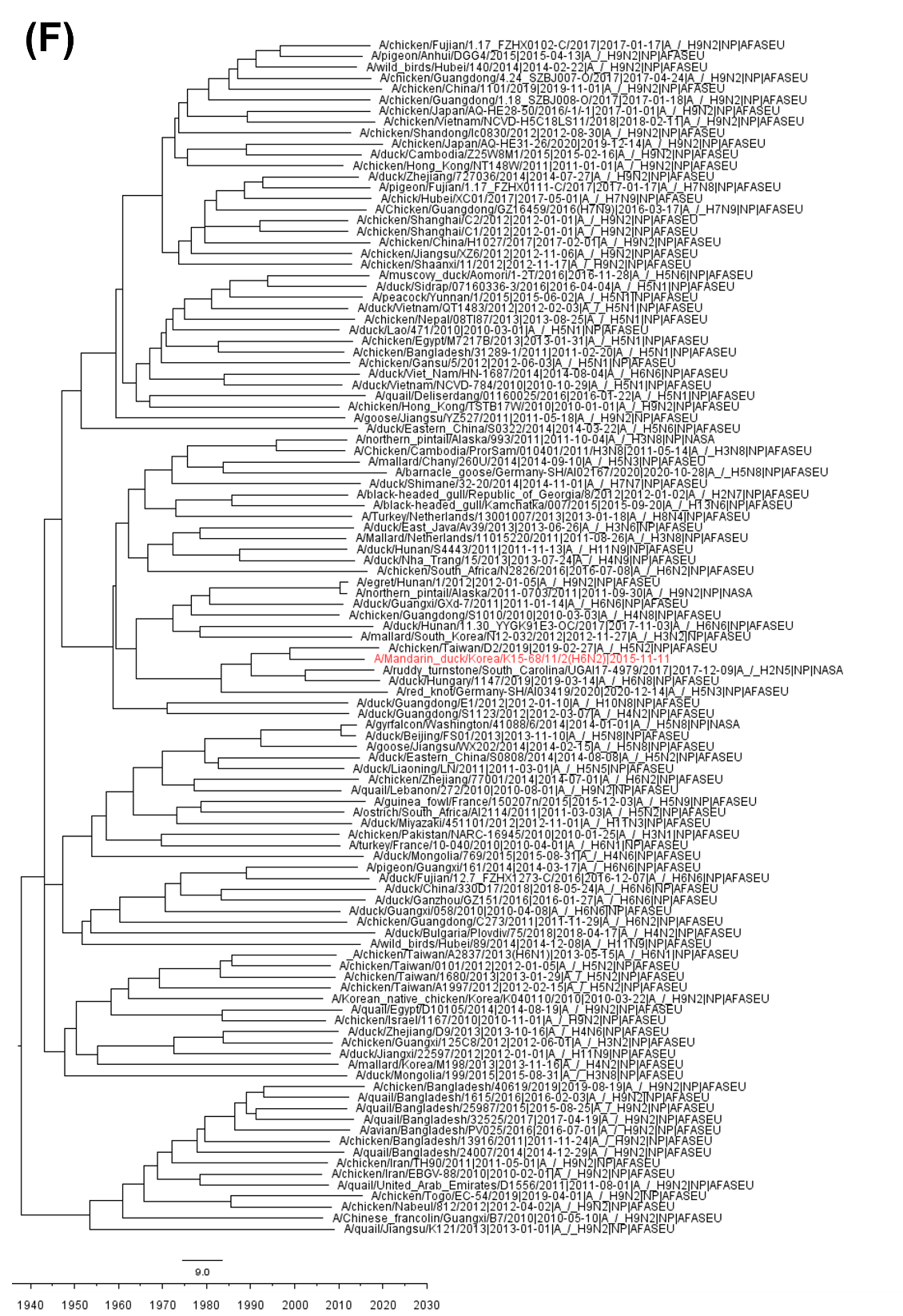


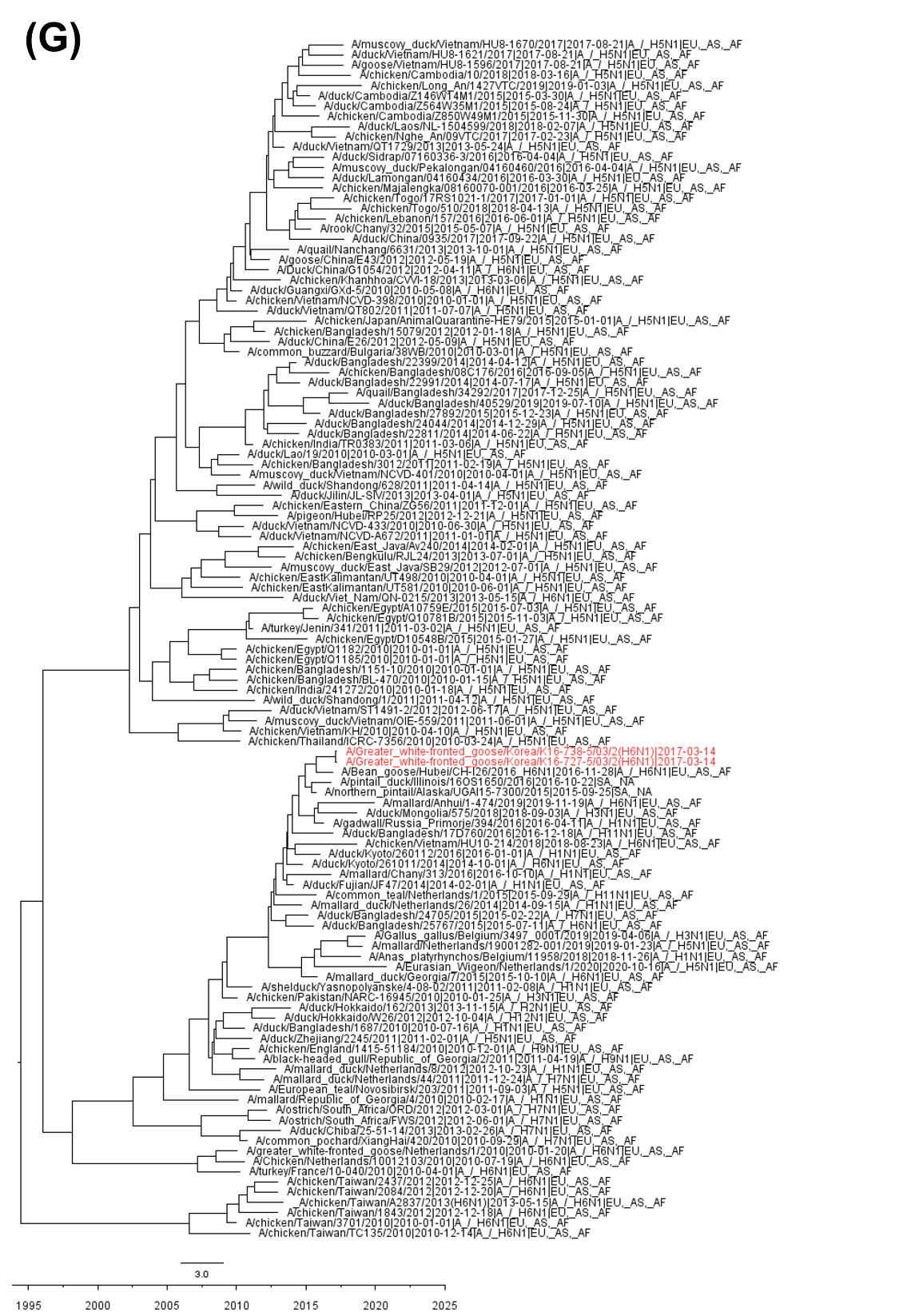


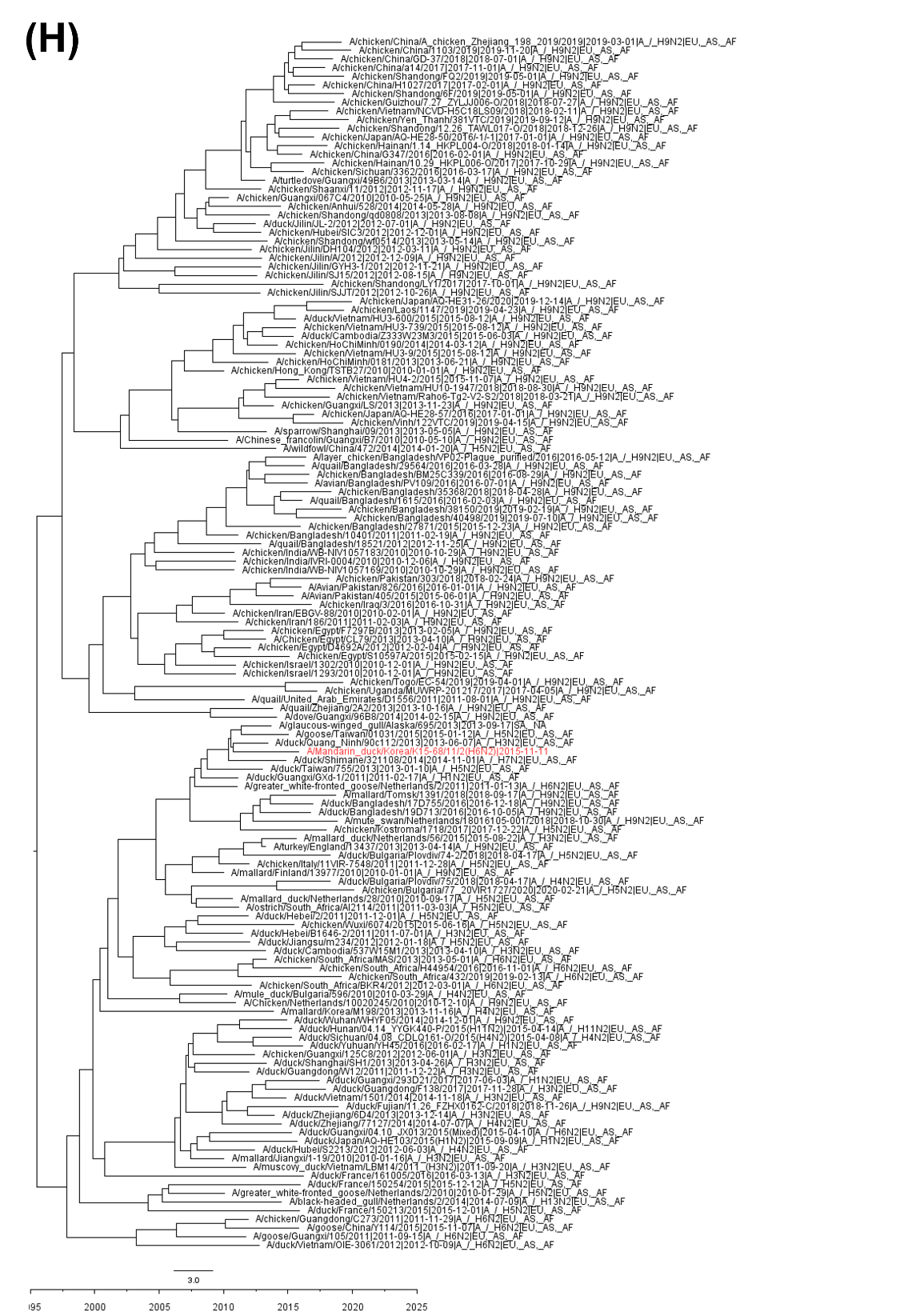


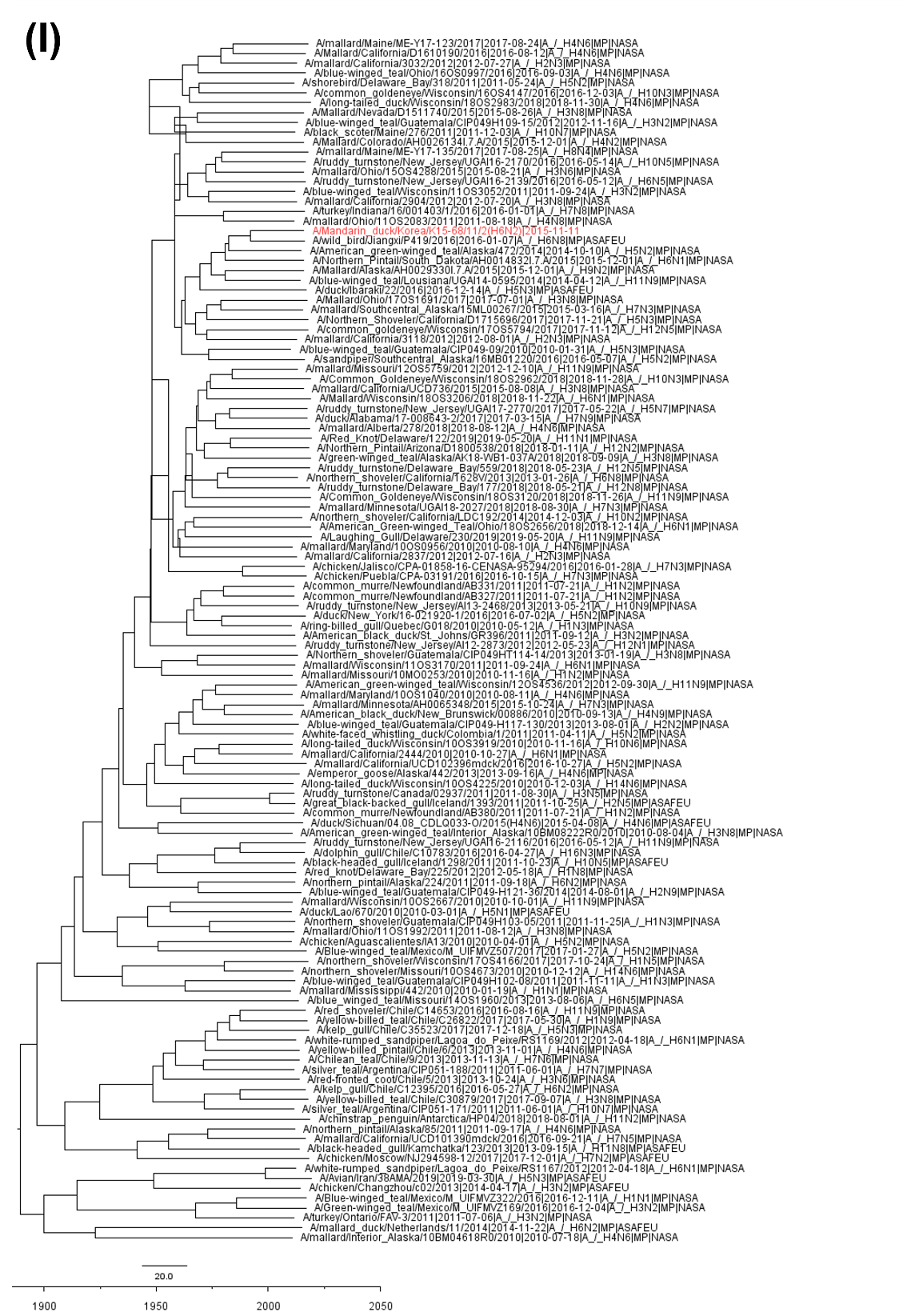


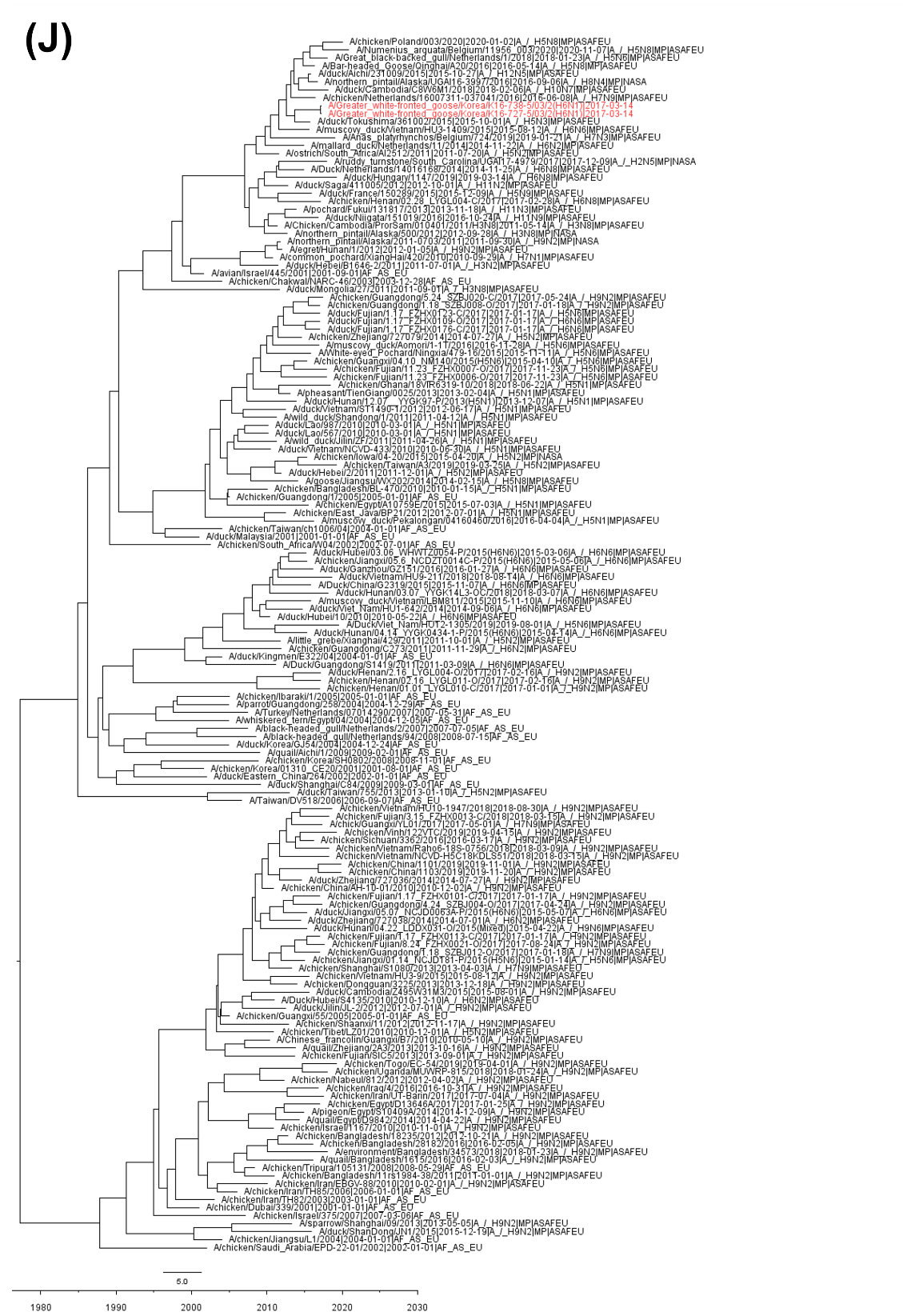


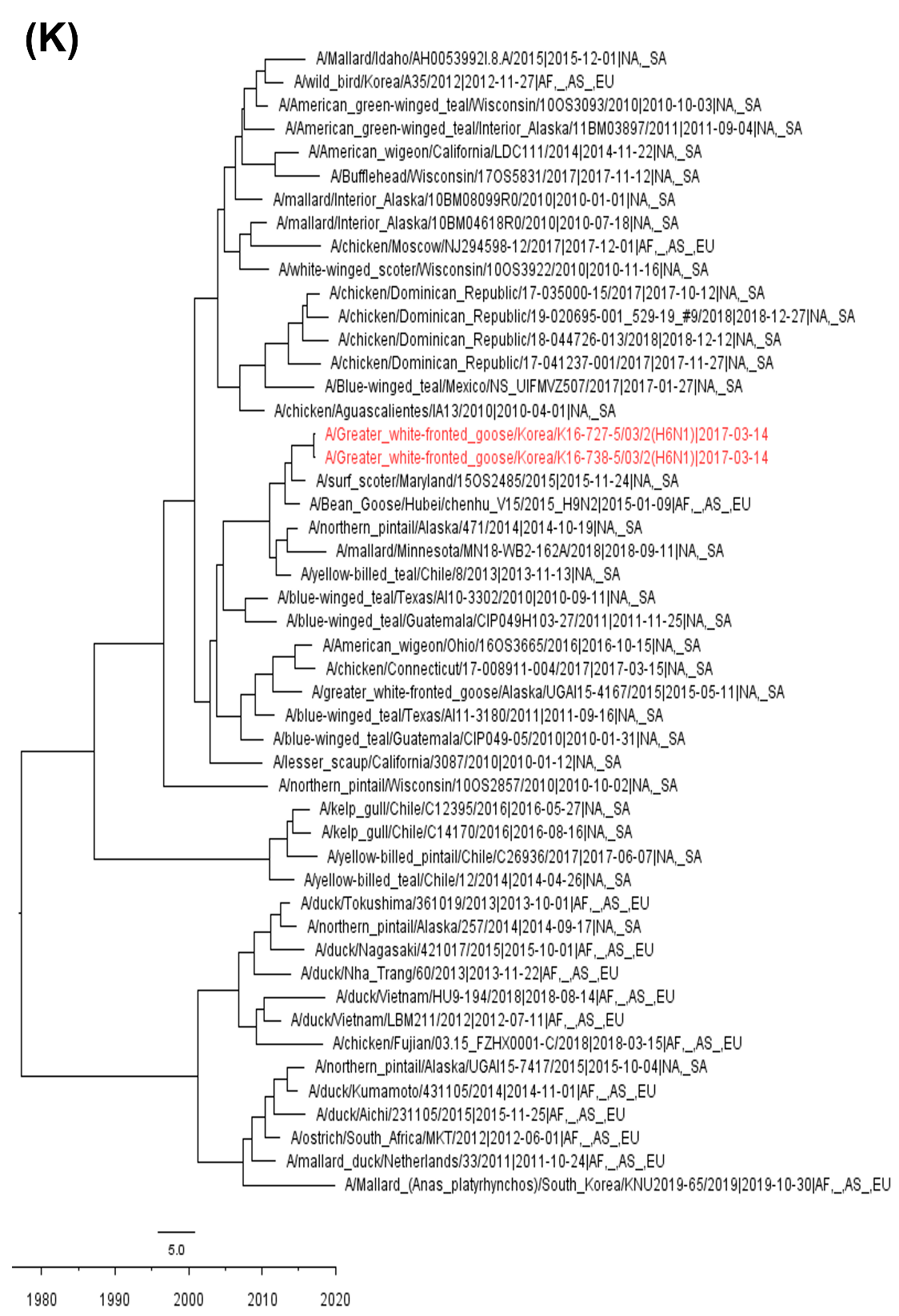


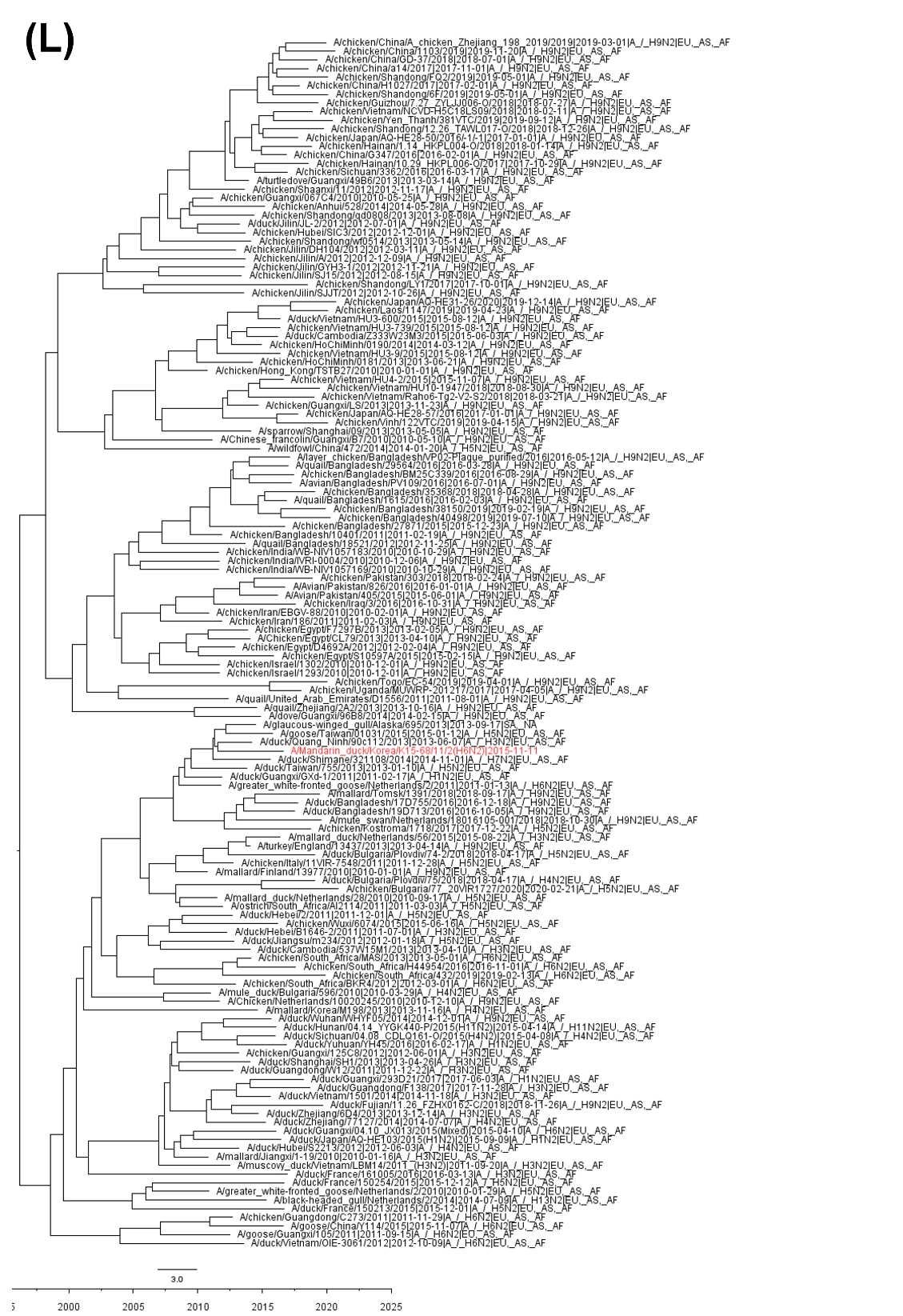
**Supplementary Figure 2.** Time scaled phylogenetic trees of cluster of AIVs genome sequences which include two AIVs isolated in this study.

Three novel viruses isolated in this study are indicated by red text. Two novel viruses that were isolated in the same date; K16-727-5, K16-738 are indicated by a red bracket. We estimated the most recent common ancestor (tMRCA) of K16-727-5 and K16-738. Horizontal bars indicate 95% Bayesian credible intervals for estimates of tMRCA. (A) Polymerase basic 2 gene; (B) polymerase basic 1 gene; (C) polymerase acidic gene; (D) hemagglutinin gene; (E) American cluster of nucleoprotein gene; (F) Eurasian cluster of nucleoprotein gene (G) neuraminidase gene subtype 1; (H) neuraminidase gene subtype 2; (I) American cluster of matrix gene; (J) Eurasian cluster of matrix gene; (K) American cluster of nonstructural gene; (I) Eurasian cluster of nonstructural gene.
